# CIRCULATING BIOMARKERS OF CELL SENESCENCE IN A RANDOMIZED TRIAL OF CALORIC RESTRICTION WEIGHT LOSS

**DOI:** 10.1093/geroni/igac059.390

**Published:** 2022-12-20

**Authors:** Jamie Justice, Iris Leng, Nathan LeBrasseur, Natalia Mitin, Stephen Kritchevsky, Barbara Nicklas, Jingzhong Ding

**Affiliations:** Wake Forest School of Medicine, Winston-Salem, North Carolina, United States; Wake Forest School of Medicine, Winston-Salem, North Carolina, United States; Mayo Clinic, Rochester, Minnesota, United States; Sapere Bio, Durham, North Carolina, United States; Wake Forest School of Medicine, Winston-Salem, North Carolina, United States; Wake Forest School of Medicine, Winston-Salem, North Carolina, United States; Wake Forest University, Winston Salem, North Carolina, United States

## Abstract

There are no circulating markers exclusive to cell senescence for clinical trials in middle-aged/older adults. In this study we evaluated T-cell expression of tumor suppressor protein p16INK4a and plasma senescence associated secretory proteins (SASP)-factors in the context of an 18-week randomized trial of caloric restriction (CR) compared to a weight stable Control. The analysis includes 55 middle-aged/older adults (75%Women; 33%African American; 57.6±5.8 years) with obesity and prediabetes. We measured mRNA expression of select senescence and apoptosis gene transcripts (p16INK4a, p21, BCL2L, BAK1) in peripheral blood CD3+ T-cells (quantitative-RT-PCR) and a panel of 27 plasma SASP proteins (Luminex/multiplex; ELISA). Weight loss in those randomized to CR was -10.8±0.9 kg compared to +0.5±0.9 kg in Control. Using mixed models, T-cell expression of senescence-biomarkers p16INK4a, p21, BCL-2L, and apoptosis-marker BAK1 were not different between CR and control at 18 weeks (time × treatment adjusted for age, race, and sex, all p>0.05). We explored associations between biomarkers with weight loss; moderate spearman correlations (rs=0.28 to 0.64) were observed between weight loss and change in plasma SASP-factors (Activin A, I-CAM, MMP1, PAI-1, TNF-RI, TNF-RII, VEGF, uPAR, p< 0.05 all), but not T-cell biomarker expression. We evaluated associations between biomarkers and found change in T-cell p16INK4a was associated with change in plasma SASP-factors, Fas (rs=0.51), osteopontin (rs=0.34), myeloperoxidase (rs=0.39), and MMP2 (rs=0.40) (CR+Control combined). Among middle-aged/older persons with obesity undergoing 18-weeks CR, changes in T cell p16INK4a expression were correlated with reductions in key SASP factors, and weight loss was associated with lower pro-inflammatory SASP profile.

